# Effects of diet and roughage quality, and period of the day on diurnal feeding behaviour patterns of sheep and goats under subtropical conditions

**DOI:** 10.5713/ajas.17.0901

**Published:** 2018-07-26

**Authors:** Mehluli Moyo, Rasheed Adekunle Adebayo, Ignatius Verla Nsahlai

**Affiliations:** 1 Animal and Poultry Science, School of Agricultural, Earth and Environmental Sciences, University of KwaZulu-Natal, Private Bag X01, Scottsville, Pietermaritzburg, 3209, South Africa

**Keywords:** Feeding Behaviour, Feed Quality, Goats, Predation Risk, Sheep

## Abstract

**Objective:**

This study investigated the effect of diet and roughage quality (RQ) on dry matter intake, duration and number of daytime and night-time eating bouts, idling sessions and ruminating activities in small ruminants.

**Methods:**

In Exp 1 and 2, RQ was improved by urea treatment of veld hay, while diet quality was improved by supplementing with Lucerne hay (Exp 3), sunflower meal and lespedeza (Exp 4), fish meal (Exp 5a), and sunflower meal (Exp 5b). In all experiments goats and sheep were blocked by weight and randomly allocated to experimental diets. Day-time (06:00 to 18:00 h) and night time (18:00 to 06:00 h) feeding behaviour activities were recorded.

**Results:**

RQ affected rumination index in Exp 1, but not in Exp 2, 3, and 5. Time spent eating and ruminating was affected by RQ (Exp 1, 3, and 4), period of day (all experiments) and their interaction (Exp 1). Intake rates (g/bout and g/min) were similar across diets. Period of day affected the duration of rumination sessions (Exp 1, 2, and 3); diet or RQ affected the duration of eating bouts (Exp 3) and rumination sessions (Exp 1 and 2). RQ had a significant effect on the duration of eating sessions in Exp 3 only, whilst period of day affected this same behaviour in Exp 2 and 3. Generally, goats and sheep fed on roughage alone ruminate at night and eat more during the day but those fed a roughage and supplemented with Lucerne hay spent more time ruminating than eating. Time spent eating and ruminating had positive correlations to crude protein and feed intake. Intake rates had strong positive correlations to intake.

**Conclusion:**

Chewing time, number of eating and ruminating sessions, and duration of eating bouts are physiologically controlled in small ruminants, though chewing time requires isometric scaling during modelling of intake.

## INTRODUCTION

Small ruminants, sheep and goats, are becoming the most important livestock species for African pastoralist communities in semi-arid and arid areas of tropical Africa [1] because they can survive in harsh conditions. Due to fluctuations in rainfall patterns, occurrence of droughts, desertification, limited crop cultivation and overgrazing, goats and sheep are increasingly facing feed shortages, especially during the dry seasons [2]. The major constraint to ruminant livestock production in semi-arid and arid areas of sub-Saharan Africa is poor nutrition due to abundance of feeds of low nutritional value, poor digestibility and scarcity of feeds [3]. It has been suggested that low levels of productivity in ruminants that graze on poor quality roughages may be a result of low feed intake. Due to the bulkiness of tropical roughages, ruminants fail to eat enough to meet their nutritional needs. Ruminants grazing on poorly digestible roughages may spend more time rechewing ingesta to render degradation more efficient, which may be viewed as an essential adaptation. However, spending more time rechewing ingesta would increase energy demand for maintenance and reduced time spent eating, resulting in animals failing to eat enough to meet requirements for maintenance and growth.

Diurnal feeding behaviour describes and encompasses activities such as time spent eating, ruminating, and idling, and the number of feeding and ruminating sessions ruminants partake on a normal circadian cycle. Duration of feeding behaviour measures may vary between individual ruminants of the same feeding type, physiological state, species, forage type, roughage quality, amount of feed allocated and probably the time of day [4]. Predator-prey interactions between artiodactyls (grazing and browsing herbivores) and carnivorous animals in an ecosystem are manifested during feeding and thus changing feeding behaviour patterns of herbivores [5]. Influences of idling, rumination and eating/grazing on frequency and amplitudes of reticulo-rumen contractions, which in turn affect fluid and solid passage rates, may influence nutrient supply, microbial protein yields and roughage intake in ruminants.

In the dry seasons, small ruminants mainly depend on poor quality crop residues such as maize stover to supplement grazing. A number of technologies have been developed to improve nutritional status of animals during the dry season, but the rate of adoption by small-scale farmers is low. These technologies include the use of cactus plant species as winter supplements, protein concentrate supplementation, treatment of hay or crop residues using lime, urea, ash or animal urine (non-protein nitrogen sources), chopping and soaking crop residues in water before offering to livestock [2]. Urea treatment of poor quality hay or crop residues has been shown to i) increase digestibility by up to 5% more than concentrate supplements, and ii) increase crude protein (CP) and energy values of forages, and generally improves the nutritional status of animals [6].

Improvement of nutritional status in goats and sheep kept by pastoralist communities’ would reduce live weight loss during the dry season necessitating increased feed intake. Reduction in live weight loss translates to a reduction in mortality of livestock, which may be viewed as a great achievement in drought stricken areas. The current authors are unaware of studies that determine how roughage intake and improvement of dietary roughage quality influence diurnal feeding behaviour in goats and sheep fed on non-supplemented urea-treated tropical veld hay, except for two studies by Chermiti et al [7] and Trach et al [8] in cattle fed on supplemented urea treated wheat and rice straw, respectively. Few studies, if any, done in subtropical and tropical Africa have evaluated all three major feeding behaviours during the day and at night at once. It is possible that diet and roughage quality affects feeding behaviour, and feeding behaviour would affect intake, so feeding behaviour should be included in mathematical models that seek to predict roughage intake in ruminant animals [9]. The aim of this study was to determine i) how improvement of hay and diet quality influences feeding behaviour and intake in goats and sheep, ii) how day-time and night-time feeding behaviour patterns vary with diet and roughage quality, and iii) whether or not there is a link between feeding behaviour patterns and feed intake. This study tested the hypothesis that improvement of roughage and diet quality has an effect on diurnal feeding behaviour patterns and intake in goats and sheep.

## MATERIALS AND METHODS

### Study site

These experimental trials were conducted with the approval of the University of KwaZulu-Natal Ethics Committee; the Animal Ethics Subcommittee (ref. AREC/072/2015M) at the University of Kwazulu-Natal’s Ukulinga Research Farm, Pietermaritzburg, in the subtropical hinterland of KwaZulu-Natal Province, South Africa. It lies at 30°24′S, 29°24′E at an altitude of 700 m. Mean annual rainfall in the study site is approximately 735 mm, falling mostly in summer, between October and April. Maximum and minimum mean annual temperatures are 25.7°C and 8.9°C, respectively. In extreme cases, summer temperatures may reach highs of above 36°C with minimum temperatures as low as 3°C at night in winter.

### Animals, housing, feeds, diets, and feeding

In Exp 1, seven adult Merino wether sheep (average initial body mass of 56±3.60 kg) were used. In one dietary treatment, roughage quality was enhanced by treating veld hay with 4% (w/w) urea for 40 days to give hay of improved roughage quality (IRQ) and the other treatment was untreated veld hay with poor roughage quality (PRQ) (Table 1). Sheep were randomly allocated to either IRQ (n = 4) or PRQ (n = 3) and given approximately 2 kg dry matter (DM) of either IRQ or PRQ veld hay at 10:00 h and 15:00 h daily for the whole duration of the trial. Chermiti et al [7] and Warly et al [10] used similar numbers of experimental animals. In Exp 2, eighteen Nguni goats were divided into two groups that comprised of nine light mass (average initial body mass of 16.94±2.51 kg) and nine heavy mass (average initial body mass of 33.6± 5.00 kg) goats. In one dietary treatment, roughage quality was enhanced by treating veld hay with 4% (w/w) urea for 20 days to give hay of IRQ, in the second treatment, veld hay was sprayed with 2.5% (w/w) urea before feeding to give semi-improved roughage quality (SIRQ), and the third treatment was untreated veld hay with PRQ (Table 1). Each group was randomly allocated to either IRQ, SIRQ, or PRQ making six goats/feed type and given approximately 2 kg DM/d of either IRQ, SIRQ, or PRQ at 08:00 h and 15:00 h daily for the whole duration of the trial.

In Exp 3, twenty-five Merino sheep (average initial body mass of 43.6±11.5 kg) were blocked by weight into five groups. Sheep in each group were randomly assigned to five dietary treatments in a completely randomised block design. These five diets were designed to provide a range of diet qualities that consisted of veld hay and Lucerne hay (LH) only, mixed in varying proportions (Table 1). Sheep were allocated approximately 2 kg DM of their diets twice (at 08:00 to 08:30 h and 15:00 to 15:30 h) daily for the whole duration of the trial. Final body mass was not determined because the trial duration was seven days only, hence body mass changes were not reported. In Exp 4, twelve Damara sheep (average initial body mass of 27.54±3.68 kg) were randomly assigned to four different dietary treatments composed of varying levels of any one of three roughage sources: maize stover at milk stage (MSM), maize stover at dry stage and grass hay. Diet qualities were varied by mixing the roughage with any one of two protein sources: cottonseed meal and Lespedeza (LSP) (Table 1) in a completely randomised design. In Exp 5, sixty four Merino lambs (average initial body mass of 22.4 ±3.65 kg) were randomly allocated to *Themeda triandra* hay offered *ad libitum*. Diet quality was improved by supplementing hay with 600 g of air-dried concentrates (Table 1). Concentrates were formulated to contain 160, 200, 240, and 280 g CP/kg and were based on either fish meal (FM) or sunflower meal (SFM). The composition of these concentrates is given in Table 2. The concentrate portion of the diet was offered in two equal portions daily between 08:00 to 08:30 h and between 15:00 and 15:30 h while the hay component was given after the allocated concentrate was completely consumed.

In all experiments, sheep and goats were allowed 14-day adaptation period to experimental diets and had >3 days to adapt to conditions in the individual crates before feeding behaviour was recorded. Sheep and goats in each study were housed in individual crates (70 cm wide, 150 cm long, and 90 cm high) with slatted wooden floors, and allowed *ad libitum* access to both roughage and water. Hay and maize stover were milled to pass through a 12 mm screen using a hammer mill (Scientec hammer mill 400, Lab World Pty Ltd, Johannesburg, South Africa). Feed left in feeders was weighed daily before new feed allocation was done. Daily feed intake was calculated by subtracting feed left from feed allocated (Intake = feed in – feed out) in all experiments, except in Exp 4 where intake and body weight changes were not determined due to the short duration of the trial. All experiments were not done concurrently.

### Behavioural assessment

Feeding behaviours assessed in each study were: duration of time spent eating, ruminating, idling whilst standing, idling whilst lying down during the day and at night. Number of feeding bouts and duration of each feeding bout during the day and at night were also determined for each study, in which the daytime period was taken to be from 06:00 to 18:00 h, and the night-time period from 18:00 to 06:00 h. A circadian assessment of feeding behaviour was conducted for Exp 1, 2, 3, and 5. In Exp 1 and 2, five closed circuit television (CCTV) cameras were used to record the feeding behaviour of sheep and goats for 24 hours a day over a 5 and 4 day period, respectively. In Exp 1 and 2, duration of activities were determined by watching the videos and recording durations and frequencies of each of these behaviours. In Exp 3, feeding behaviour was recorded on 3 different days for periods of 24 hours at a time. Each 24-hour period was divided into 1 h long periods, which in turn were divided into five-minute segments, and the activity of individual sheep observed and recorded. In Exp 4, an observer, positioned on a spot where all sheep could be seen, recorded feeding behaviour without disturbing animals. Before any visual observation of sheep commenced, sheep were given feed *ad libitum*. Use of once-off feeding was adopted so as to have disturbance-free sessions when feeding behaviour was recorded. Activities were recorded at 2-minute intervals for 10 hours for 3 consecutive days. In Exp 5, each 24-hour day was divided into 8 periods of three hours each during which two enumerators (each assigned to specific animals) sat on either sides of the pens and recorded the activity of sheep every two minutes.

### Chemical analyses and design of experimental feeds and diets

Moisture, dry matter, organic matter and ash were analysed using the procedures described by the Association of Official Analytical Chemists [11]. Nitrogen content was determined using the LECO TruSpec nitrogen analyser (LECO FP2000, LECO, Pretoria, South Africa). The CP content was calculated by multiplying the nitrogen content by a factor of 6.25 (CP = nitrogen content×6.25). Neutral detergent fibre (NDF) and acid detergent fibre (ADF) were analysed using ANKOM A220 fibre analyser (ANKOM Technology, New York, USA). Hemicellulose content as determined by subtracting ADF content from NDF content (Hemicellulose = NDF – ADF). Crude fat content was determined using the Soxhlet method on the Soxhlet Buchi 810 fat analyser (Soxhlet Buchi, Flawil, Switzerland).

### Statistical analysis

Effects of roughage and diet quality on intake (except Exp 4) and feeding behaviour were statistically analysed using the general linear model (GLM) procedure of SAS 9.3 software (SAS Institute Inc., Cary, NC, USA). The GLM procedure was also used to determine the effect of roughage quality, period of day, and roughage quality and period of day interactions on feeding behaviour parameters (Exp 1, 2, 3, and 5). The Student-Newman-Keuls (SNK) test was used to separate sample means that were significantly different from each other at p<0.05. Initial body mass (BM) was taken as a covariate. The experimental model for feeding behaviour was as follows: FB_ijkl_ = μ+R_i_+P_j_+(R×P)_ij_+BM_k_+e_ijkl_, where: FB = feeding behaviour (eating time, ruminating time, idling time whilst standing, idling time whilst lying), μ = overall mean, R_i_ = effect of roughage or diet quality, P_j_ = effect of period of the day (j = day; night), (R×P)_ij_ = effect of roughage quality and period of day interactions and e_ijkl_ = experimental random error.

The Pearson correlation of all the continuous independent variables (CP, NDF, ADF, and hemicellulose) was used to select variables tested as covariates. The correlation between these variables was such that NDF and ADF could be used singly and CP and hemicellulose as a pair. Judging from the error term, the accepted model was the one with CP content. A meta-analyses of feeding behaviours from all 6 studies was done and data were analysed using the mixed model regression procedure [12]. A model with discrete predictor variables (ruminant species, period of day and ruminant species×period of day interactions) and continuous predictor variables (CP content of diets) was used. These respective predictor variables were considered as fixed effects. Study× straw quality (whether roughages were treated with urea or not) interactions were considered as random effects. For the discrete predictor variable (ruminant species, period of day and ruminant species× period of day interactions), the following model was applied: FB_ijklm_ = μ+R_i_+P_j_+(R×P)_ij_+ CP_k_+SQ_l_+e_ijklm_; where Y_ijklm_ = feeding behaviour (times spent eating and ruminating, number of eating and ruminating bouts, and duration of eating and ruminating bouts), μ = overall mean, SQ_l_ = random effect of study× roughage quality, R_i_ = fixed effect of ruminant species (i = sheep; goats), P_j_ = fixed effect of period of the day (j = day; night), (R×P)_ij_ = fixed effect of ruminant species×period of day interactions, CP_k_ = fixed effect of crude protein content and e_ijklm_ = residual error. Data were weighted by the number of animals in each study and the standard errors of the mean [12]. Least square means were used to compare the differences among means in the case of discrete predictor variables. The probability threshold for significance of fixed and random study effects was considered at p<0.20 as suggested by Sauvant et al [12].

## RESULTS

In Exp 1, DM and NDF intake was higher for sheep fed on IRQ compared to sheep fed PRQ. Time spent ruminating per unit of dry matter and NDF intake were significantly higher for sheep fed PRQ compared to those fed IRQ, while average dry matter intake rates per unit time and feeding bout were similar for these two roughage qualities (Table 3). On an average day, sheep fed on IRQ spent 19%, 34%, and 47% whilst those fed on PRQ spent 13%, 45%, and 42% of the day eating, ruminating and idling, respectively (Table 4). There was great variation in daytime and night-time feeding behaviour patterns between and within each dietary treatment. Ruminating time and duration of ruminating bouts were longer for sheep fed PRQ compared to those fed IRQ. Sheep fed IRQ spent significantly more time eating during the day, but less time ruminating during the day and night compared to those fed on PRQ. Irrespective of roughage quality, sheep spent significantly more time eating during the day than at night, but less time ruminating during the day than at night. Ruminating sessions were longer at night than during the day.

Number of ruminating bouts were higher at night than during the day, while sheep visited feeding troughs 3 times more during the day than at night. Sheep spent more time idling whilst standing during the day than at night. Time spent idling whilst lying was greater at night than during the day. At night, sheep fed IRQ spent significantly more time idling whilst lying than sheep fed on PRQ. Sheep fed IRQ chewed less, however, sheep fed PRQ chewed more at night and less during the day than those fed on IRQ. Sheep fed PRQ lost 0.14 kg/d more than those fed on IRQ.

In Exp 2, unexpectedly, average dry matter intake, NDF intake, ruminating indices (dry matter and NDF), average dry matter intake rates per unit time and feeding bout were similar for all 3 roughage qualities in goats (Table 3). On an average day goats spent 20% eating, 28% ruminating and 52% idling (IRQ), 20% eating, 25% ruminating and 50% idling (SIRQ), but spent 17% eating, 21% ruminating and 62% idling (PRQ) (Table 5). Roughage quality had no effect (p>0.05) on all feeding behaviour parameters except for duration of ruminating bouts. Period of day significantly affected all feeding behaviour parameters except for time spent chewing. Goats spent more time eating and standing while idling during the day than at night, but spent more time ruminating at night than during the day. Number of eating bouts were greater during the day than at night, however, the number of ruminating bouts were greater during the night than during the day. Eating bouts were longer during the day than at night across all roughage qualities, with an opposing trend observed for the duration of ruminating bouts, which were longer at night than during the day. Goats spent more time lying at night than during the day. Goats fed PRQ lost 0.06 and 0.04 kg/d more than those on IRQ and SIRQ, respectively.

In Exp 3, diet quality had no effect on average dry matter intake, ruminating indices (dry matter and NDF), NDF intake and average dry matter intake rates per unit time and feeding bout (Table 3). On an average day sheep spent 25% eating, 39% ruminating and 36% idling (100% PRQ); 23% eating, 42% ruminating and 35% idling (75% PRQ); 21% eating, 38% ruminating and 41% idling (50% PRQ); 18% eating, 40% ruminating and 42% idling (25% PRQ); 19% eating, 37% ruminating and 44% idling (100% LH) (Table 6). Diet had an effect on eating and chewing time, time spent idling whilst standing, and duration of eating bouts. Increasing levels of LH decreased eating time during the day and at night relative to 100% PRQ. Similarly, overall chewing times decreased during the day and at night relative to 100% PRQ. Differences in time spent idling while standing did not follow a consistent trend with increasing Lucerne content of diets during the day, but increased gradually at night relative to 100% PRQ. Period of day influenced all feeding behaviours measured, whilst time spent chewing and number of ruminating bouts were significantly affected by diet and period of the day interactions. Sheep spent more time eating during the day than at night, but surprisingly, spent more time ruminating during the day than at night and less time idling while standing at night than during the day. Eating bouts were longer during the day than at night, and so were the number of eating bouts. Ruminating sessions were surprisingly longer during the day than at night, although the frequency of ruminating bouts was greater at night than during the day. Sheep spent more time lying at night than during the day.

In Exp 4, on an average 10 h daytime period sheep spent 36% eating, 22% ruminating, and 42% idling (MSM+40% SFM); 48% eating, 27% ruminating and 25% idling (40% LSP); 26% eating, 19% ruminating and 55% idling (60% SFM) and 32% eating, 23% ruminating and 45% idling (GH+40% SFM) (Table 7). Times spent eating were statistically similar for all diets, except for sheep fed on 40% LSP that ate longer than those fed on 60% SFM diet. Supplementation with 40% SFM compared to 40% LSP when MSM was the main roughage source decreased chewing time. Except for sheep fed MSM+ LSP which spent more time eating per session compared to other dietary treatments, durations of eating bouts were similar across 3 diets (p>0.05). When MSM was the main roughage source, replacement of 40% SFM with 40% LSP increased duration of ruminating sessions.

In Exp 5a, diet quality had no effect on intake, rumination indices and intake rates (Table 3). On an average day, sheep spent 15% to 17% eating, 34% to 39% ruminating, 45% to 51% idling across all levels of FM supplementation (Table 8). Eating bouts were longer during the day than at night for sheep fed on 16%, 20%, 24%, and 28% FM supplement. However, ruminating sessions were longer at night than during the day, while the number of ruminating bouts were greater during the day than at night. Sheep supplemented with FM gained on average 0.185 kg/d in body mass.

Generally, sheep spent 15% to 16% eating, 32% to 33% ruminating, 50% to 52% idling across all levels of SFM supplementation (Exp 5b). Diet quality had no effects on all diurnal feeding behaviour parameters in sheep fed increasing levels of SFM supplement (Table 8). Period of day affected time spent ruminating and eating, duration of eating and ruminating sessions, and number of ruminating sessions. Eating sessions were longer during the day than at night, while ruminating bouts were longer at night than during the day. Unexpectedly, the number of ruminating bouts were greater during the day than at night. Also, sheep spent more time eating during the day than at night, and spent more time ruminating at night than during the day. Times spent idling and chewing were evenly distributed throughout the day and at night. Generally sheep supplemented with SFM on average gained 0.136 kg/d in body weight.

Overall, time spent eating between goats and sheep was similar, however, sheep spent more time eating during the day and at night compared to goats (Table 9). Both sheep and goats ruminated more at night than during the day, although sheep spent more time ruminating than goats irrespective of period of the day. Number of eating and ruminating bouts were similar for both sheep and goats. Sheep spent more time chewing than goats. Eating bouts were longer during the day than at night for both sheep and goats, while an opposing trend was observed where ruminating bouts were longer at night.

Dry matter intake was higher for sheep compared to goats (Table 10). Effects of variable CP in the diets seemed not to have an effect on intake, rumination indices and intake rates. Goats had lower overall intake rates (g/min) than sheep, while overall intake rates per feeding bout were similar for both ruminant species.

All feeding behaviours had significant positive correlations to intake (Table 10). Time spent chewing and ruminating have significant (p<0.05) correlations to intake (r ≈ 0.5). There was a significant (p<0.05) correlation between time spent ruminating and eating (r>0.5) to time spent chewing. Time spent eating was not (r<0.5; p>0.05) correlated to time spent ruminating. Intake rates had a correlation to intake (r>0.7; p<0.0001). Surprisingly, the major feed attributes (NDF and ADF) though correlated between them had positive and significant (p<0.005) correlations to intake. The CP content had significant correlations to time spent eating and ruminating, and intake rates.

## DISCUSSION

Diurnal feeding behaviour in ruminants is not seen as a way of predicting feed intake, but rather as a way of explaining intake [4]. The influence of diet and roughage qualities on eating, ruminating and idling behaviour, and roughage intake in ruminants fed low quality roughages in subtropical and tropical Africa have been overlooked. Thorough understanding of intake in ruminants involves studying the major aspects of feeding behaviour; eating, ruminating, and idling [13]. Unexpectedly goats fed IRQ, SIRQ, and PRQ (Exp 2) ate statistically equal amounts these feeds, though the tendency was IRQ>SIRQ> PRQ, confirming that urea treatment is more than just additional CP. Goats are more likely to consume large proportions of leafy parts than sheep given their inclination to select. Sheep and goats are sensitive to the four primary tastes: sweet, salty, bitter and sour [14] and odoriferous compounds [15]. Improvement of roughage quality using urea may have altered one of the four tastes leading sheep to consume more (Exp 1), but not goats (Exp 2). Urea-treated hay is characterised by a strong pungent odour, which is expected to deter animals from eating [16]. However, it seems that sheep (Exp 1) preferred eating more of the hay with a pungent odour, but not goats (Exp 2). Sensory perception of these compounds might be different in goats and sheep. Thus, research should assess whether the pungent odour, colour and taste of urea treated hay is partly responsible for changing intake in sheep (Exp 1), goats (Exp 2), and cattle [16], respectively. Furthermore, the effect of scent on feeding behaviour and intake warrants research. Unexpectedly, improving diet quality by increasing levels of LH, and supplementation with fish and SFMs did not alter feed intake in sheep.

In this study, sheep and goats across all experiments maintained statistically similar intake rates (g/bout), thus differences in feed intake in any of the experiments would only be due to differences in bite frequency and bite mass/size. Unfortunately, bite frequency and size were not measured in this study. Similar conclusions were drawn by Penning et al [17] and Rutter et al [18]. Rutter et al [18] experimenting on sheep fed on rye grass and clover, found that dry matter intake rates were similar between dietary treatments. This suggests that under any dietary condition intake rates are largely under the control of the animal’s physiological status in ruminants fed indoors. Under grazing conditions, intake rates are affected by feed factors such as leaf size and sward height [19], and sward density, which are non-existent indoors. Ruminants reduce intake rates and increase eating time, and vice versa, so as to maintain desired feed intake levels through management of grazing or eating time [20]. This motivation to eat depends on the animal’s needs and, day and night time feeding patterns. Hay treatments used in our study (Exp 1 and 2) were of the same grass species with their quality differing as a result of treatment with urea only, although, there was a possibility of slight differences in organoleptic properties between these treatments, intake rates for IRQ, SIRQ, and PRQ hays were expected to be similar. Intake rates for feeds and diets in this study are similar to those obtained by Baumont et al [21] with sheep fed LH, but different from those of Dominigue et al [22]. Initial intake rates accounted for most differences in daily feed intake [21], but unfortunately it was not measured in these studies. So, from the above, it seems possible that roughage intake may be controlled using two methods that are antagonistic: by either increasing eating time whilst maintaining constant intake rates [20], or by increasing intake rates whilst maintaining constant eating time. Factors influencing the adoption of any one of these intake control mechanisms warrants further study. Differences in rumen fill levels at any given time between sheep and goats on all treatments may govern feed intake by partially controlling intake rates and time spent eating. The lower the rumen fill levels the more the receptive space in the rumen to accommodate more feed and eventually the greater the intake rate and time spent eating. Rumen fill levels and fatigue as a result of increasing eating time to compensate for low intake rates can barely be used to explain the overall time spent eating and ultimately intake in ruminants [17]. Additionally, most studies, including the current study have failed to account for the effect of the number of hedonic feeding sessions and their duration as a factor that increases time spent eating. Studies have reported different frequencies of small meals across different types of hay, and although durations of small meals were not reported, small meals increased time spent eating by sheep [21] and increased feed intake in goats [13]. The challenge lies in setting a time range for feeding bouts to be classified as hedonic. It is worthwhile to determine how roughage and diet quality, and period of the day influence frequencies and duration of hedonic bouts in pen fed and grazing ruminants.

Generally, ruminants spend more time ruminating compared to eating. This is in line with our findings from all experiments, although findings by Abijaoude et al [13] have shown that there is a tendency to spend more time eating than ruminating in goats fed on different diets. Daily time spent ruminating, and the duration of ruminating sessions generally increased for sheep fed hay of poor quality (Exp 1) and sheep fed increasing levels of SFM and FM (Exp 5), which is similar to results by Jalali et al [23] in sheep, goats and llamas. In Exp 2, eating time in goats was not a function of roughage quality, which is different for sheep fed same feeds (Exp 1) and sheep fed increasing levels of Lucerne (Exp 3). These results suggest that eating time in goats is based on the desire to eat or hedonic eating. As anticipated, time spent eating and chewing decreased with increased levels of LH (increased diet quality). Overall chewing time in goats (Exp 2), number of eating and ruminating sessions (all experiments), and duration of eating bouts in sheep (Exp 1) were not affected by diet quality. This may suggest that these are physiologically controlled behaviours in goats and sheep. No significant changes in ruminating time as a result of improving feed or diet quality have been reported in cattle fed urea treated hay [8], in agreement with results for goats (Exp 2) and sheep (Exp 3 and 4). In support of our findings (in Exp 1), Chermiti et al [7] reported that cattle spent more time ruminating per unit intake of untreated straw (PRQ). Urea treatment of forages breaks lignocellulose bonds between plant cells reducing their physical strength [24]. Urea-treated hay is expected to be soft and easy to chew, thus reducing ruminating time. Improvement of hay quality using urea treatment reduced ruminating index [7,8], however, not in goats (Exp 2).

Unexpectedly, ruminating indices decreased with increasing levels of SFM, and were lower for SFM compared to FM (Exp 5). Given the high NDF content of SFM compared to FM, it was expected that sheep would spend more time ruminating per unit intake of SFM than FM. It could be that the size and fragility of fibre in sunflower facilitate rumen passage rate of these particles. Ruminating indices in Exp 1 and 2 were approximately between 2–5 times as high as for cattle fed on urea treated straw. These results suggest that goats and sheep would be less efficient in chewing the cud than cattle, probably due to a smaller total surface area of the molars than cattle as tooth surface area is isometrically scaled to BW^0.67^ [25]. Chewing efficiency in mammalian herbivores is influenced by morphological adaptations in the dental design [26]. Data from Kaske et al [27] suggests that sheep need 10-fold more chews per unit of NDF intake to equal efficiency in ruminating cattle, hence, goats and sheep are likely to spend more time rechewing digesta per unit DM and NDF intake. All but one of the ruminating times reported in this study are consistent with Welch’s [28] proposed physiological daily rumination upper limit of 600 min/d. Daily ruminating time in Exp 1 was above the proposed physiological upper limit for sheep fed PRQ, which is similar to findings by Deswysen and Ehrlein [29] in sheep fed silage (607 and 653 min/d), Kaske and Groth [30] in pregnant ewes (679 min/d) and Minervino et al [31] in sheep fed coast-cross hay (668 min/d). There are general suggestions that high levels of feed intake increase time spent ruminating. It is possible that over time ruminants have adapted to storing more roughage in the rumen when consuming poor quality roughages in the tropics [32]. Hence, sheep in Exp 1 spent more time ruminating digesta of a diet that was consumed in lower quantities. It is clear that longer ruminating times were a result of low roughage quality but not high intake levels, thus rumination time is a function of roughage quality rather than just the level of intake. However, correlation results suggest that rumination time is a positive function of intake (Table 9) and is likely to increase with rumen ‘fill’ which is higher in animals after prolonged adaptation to roughage diets [32]. Observed rumen fill levels (kg fibre/100 kg weight) of greater than 2.2 were seen in goats [33] when 1.7 is expected for temperate ruminants [34]. A value greater than 1.7 should be applied to ruminants fed on tropical roughages in Africa.

Due to the impending reduction in roughage quality of most tropical grasses as a result of climate change, ruminants will likely adapt to improve utilisation of poor quality roughages by storing more roughage in the rumen and increasing rumination time. In semi-arid, low rainfall areas of Africa there are very short growth periods for grasses causing early maturity. Rapid attainment of maturity would reduce lignification and increases CP levels slightly in grasses. Based on the positive relationship between CP content and intake rate (g/min) obtained in this study, CP may play a role in influencing feeding behaviour through intake rate. The generally low CP levels of mature tropical grasses led to goats maximising nutrient intake rates during the wet seasons when feeds of high nutritional quality (high CP levels) are abundant so as to build up enough reserves to survive the dry season [35]. It is possible an increment in the CP content and a decrease in NDF (Exp 3) would increase the intensity of microbial activity, depress the pH [36] thus reducing the need for extended rumination times for optimal nutrient extraction. Effects of CP levels on feeding behaviour raised in the above discussion are strengthened based on the Pearson correlation of CP with time spent eating and ruminating, which are confounded by digestion rate and animal species, hence, more studies are needed to ascertain the extent to which different CP levels in feeds would affect feeding behaviour in ruminants under grazing conditions in tropical Africa.

The absence of differences in the daily duration of eating sessions, and number of eating and rumination periods across dietary treatments is in line with a general consensus that the number of eating and rumination periods are not affected by roughage quality and kind of feed [10,21]. Where animals have similar daily feed intake levels, the individual number of eating sessions may vary up to fourfold [4]. Control of the number of eating sessions may be under biological control as determined by the desire to eat.

Photoperiod played a huge role in influencing daytime and night-time feeding behaviours measured in the current study, except for the duration of eating sessions and time spent idling whilst lying in sheep (Exp 1), chewing time in goats and sheep (Exp 2 and 5, respectively), and idling time in sheep (Exp 5). The effect of period of day on the number of eating and ruminating sessions, time spent eating and ruminating, and duration of rumination sessions only strengthens the fact that sheep and goats fed only on roughage diets eat during the day and ruminate at night. Ruminants fed varying levels of roughage and concentrate may not follow a similar trend, as shown in sheep (Exp 3) that ruminated more during the day than at night when given increasing levels of LH. Instead, goats evenly distribute number of meals between the day and night so as to avoid digestive and metabolic upsets such as acidosis [13] when fed diets containing concentrates, but the number of meals were higher during the day than at night when fed a roughage alone (Exp 2). In this study, goats and sheep have distinct feeding behaviours when fed on poor quality roughages. Sheep spent more time chewing and tended to spend less time eating poor quality hay. On the other hand, goats are selective of leafy parts on grass stalks but sheep are less selective consuming more of the fibrous grass stalks. Leafy parts are more digestible than stalks, hence requiring less time ruminating in goats than in sheep. Selective feeding on poor quality roughages by goats extends the need for more time spent eating to achieve adequate intake to meet nutritional needs compared to sheep. Time spent eating at night accounts for approximately 10% to 15% of the total daily eating time [37], which is fairly lower than 19% to 30% (Exp 1, 2, and 5) and 39% to 42% (Exp 3) reported in this study.

It is doubtless that goats and sheep in this study followed a strict circadian rhythm of idling whilst standing, ruminating and eating. The concept of predation and instinct may explain some of these adherences to strict circadian cycles. Risk of predation is greater during eating than ruminating because animals maintain poor levels of vigilance when eating as their heads are positioned downwards [18]. Due to instinctive fear of predation, ruminants will alter their feeding behaviour patterns with respect to period of the day, but maintain a balance between levels of vigilance in each feeding behaviour to the perceived risk status of that period of day. As a result, ruminants will spend more time grazing or eating during the day than at night, and spend more time ruminating than eating at night as shown in this study. To make up for the reduced vigilance on the threat of predation posed by spending more time eating during the day, ruminants may have to spend more time idling whilst standing during the day than at night. Idling whilst standing during the day balances the total time of engaging in a behaviour that maintains good levels of vigilance during the day. Sheep (in Exp 3) displayed a unique way of reducing the perceived risk of predation. Ruminating time, number and duration of rumination sessions were greater during the day than at night and so were eating time, the number and duration of eating sessions. This means that these sheep are aware that predation risk is higher at night and hence did everything during the day. As such they spent more time idling whilst standing at night than during the day so as to stay vigilant over the night. However, idling time lying was greater at night than during the day. This means that at night these sheep spend more time lying and standing than during the day. In Exp 5a and b, daytime and night-time behaviours only peculiar to sheep supplemented with increasing levels of protein concentrates was observed in this study. Frequencies of ruminating sessions were greater during the day than at night with number of eating bouts independent of period of the day. This suggests that sheep took regular breaks to ruminate so as to increase vigilance levels following eating during the day. This may be observed by the small difference between times spent ruminating at night and during the day (<8 min across all diet qualities). The concept on the role of idling behaviour in relation to maintenance of vigilance toward predation risk are still not well documented. More research is needed to clarify issues on the circadian control of feeding behaviour patterns in different ruminant species and genotypes that co-exist and graze tropical grasslands in relation to the concept of predation.

Consistent with our findings (from Exp 1 alone), Baumont et al [21] reported significant effects of type of hay ×period of day interactions on time spent eating and ruminating. Von Engelhardt et al [38] and Minervino et al [31] also reported similar results for ruminating activities in camels and sheep over various diet qualities, although studies by Hailu (2003 cited by Von Engelhardt et al [38]) on camels showed that rumination activities were evenly distributed throughout the day and night. Minervino et al [31] observed higher rumination activity occurred during the day than at night (similar to results from sheep in Exp 3) and eating times were evenly distributed throughout the day and night for sheep fed high concentrates diets. For some mysterious reason, duration of eating bouts was not affected by diet quality (all experiments) nor by period of day (Exp 1). These findings tend to suggest the existence of a physiological limit for eating time per session, irrespective of diet quality and period of day. Fatigue due to exceedingly long hours ruminating per day was expected to result in longer time being spent idling whilst lying in sheep fed PRQ hay (Exp 1). Contrary to these expectations, and similar to findings by Rutter et al [18], sheep in our study increased ruminating time at the expense of time spent idling. Chewing time was evenly distributed during the day and night within each treatment. An absence of the influence of period of day on chewing behaviour in sheep and goats (Exp 1, 2, and 5) strongly indicates that chewing time is mainly a function of roughage quality, although results from sheep (Exp 3) showed that chewing time is dependent on roughage quality, period of day and their interaction. Genotype, season and daytime affected feeding behaviour of goats and sheep on the rangeland, and time spent grazing was strongly influenced by seasonal variations [39]. It would be worthwhile to determine how diurnal feeding behaviour patterns (eating, ruminating, and idling) of goats and sheep are affected by season of the year, where the lengths of the photoperiods and scotoperiods are different, in tropical Africa.

As expected, positive correlations of times spent eating, ruminating and chewing, and intake rates to intake suggest that there are possibilities of using feeding behaviour to predict intake (Table 11). Based on these correlation results, time spent eating and chewing, and intake rate (g/min) are behavioural parameters to include in intake prediction models.

Due to a low nitrogen (CP) content (Exp 1 and 2) and less time spent eating by sheep fed PRQ (Exp 1), feed intake was low, resulting in goats and sheep failing to eat enough feed to meet their nutritional needs. Consequently, back-fat reserves would have been mobilised to supply energy for maintenance cost due to increased time re-chewing PRQ in Exp 1. Although sheep fed IRQ lost just little weight, they barely managed to maintain themselves partly due to higher CP levels and improved digestibility. Sheep supplemented with protein concentrates recorded body mass gains. Protein content in the diets was in excess of maintenance requirements.

In summary, chewing time, number of eating and ruminating session, and duration of eating bouts are physiologically controlled in small ruminants, though chewing time requires isometric scaling during modelling of intake. Idling time may still be controlled by the perceived fear of predation. Goats and sheep fed on roughage alone ruminate at night and eat more during the day, but sheep fed a roughage and supplemented with LH spent more time ruminating than eating. Time spent eating, ruminating and chewing were affected by diet quality and time of the day. Improved feed quality increased eating time during the day but not at night. Reducing roughage quality tripled the difference in daytime chewing the curd at night.

## CONCLUSION

Roughage intake is limited as a result of increased rumination time of low quality roughages. Chewing time, number of eating and ruminating session, and duration of eating bouts are physiologically controlled in small ruminants, though chewing time requires isometric scaling during modelling of intake.

## IMPLICATIONS

New intake models may incorporate diet or roughage quality and period of the day as major predictor variables for feeding behaviour. Prediction of feeding behaviour in ruminant animals may be used to improve prediction power of models that seek to predict digesta passage rate through the rumen provided that feed intake, frequency of rumen contractions and the amounts of digesta that passes out at each contraction are known. A simultaneous evaluation of roughage intake, rumen fill levels, passage rates, digestibility and feeding behaviour are central to our understanding of the evolutionary adaptation of ruminant digestive physiology. Mathematical models that seek to predict roughage intake in sheep and other ruminant animals should incorporate factors that affect intake rates and time spent eating, ruminating and chewing.

## Figures and Tables

**Table 1:** Chemical composition and design of treatment diets

Items	Chemical composition (g/kg DM)						

	DM	CP	NDF	ADF	HEM	Ash	CF
Experiment 1							
IRQ	923	91	746	417	330	86	12
PRQ	926	40	735	391	344	67	13
Experiment 2							
IRQ	904	76	723	632	91	70	12
SIRQ	920	48	723	592	131	83	11
PRQ	923	20	735	581	154	89	13
Experiment 3							
100% PRQ	916	46	787	527	260	60	27
75% PRQ+25% Lucerne hay	911	81	758	534	224	66	23
50% PRQ+50% Lucerne hay	908	116	729	541	188	72	20
25% PRQ+75% Lucerne hay	904	150	700	549	151	78	16
100% Lucerne hay	900	185	672	556	116	84	12
Experiment 4							
60% MSM+40% SFM	896	192	455	279	176	69	16
60% MSM+40% LSP	901	77	544	353	191	68	19
40% MSD+60% SFM	910	235	456	273	183	69	16
60% GH+40% SFM	919	179	532	324	209	64	27
Experiment 5a							
*Themeda triandra* hay	931	61	733	440	293	40	12
TTH+16% FM concentrate	902	111	366	238	128	59	31
TTH+20% FM concentrate	903	134	365	237	128	68	29
TTH+24% FM concentrate	906	162	382	247	135	72	32
TTH+28% FM concentrate	907	183	383	247	136	76	36
Experiment 5b							
TTH+16% SFM concentrate	908	112	401	257	144	52	32
TTH+20% SFM concentrate	911	134	422	269	153	60	34
TTH+24% SFM concentrate	911	157	447	282	165	66	36
TTH+28% SFM concentrate	916	179	471	296	175	67	38

DM, dry matter; CP, crude protein; NDF, neutral detergent fibre; ADF, acid detergent fibre; HEM, hemicellulose; CF, crude fat; IRQ, improved roughage quality; PRQ, poor roughage quality; SIRQ, semi-improved roughage quality; MSM, maize stover at milk stage; LSP, lespedeza; MSD, maize stover at dry stage; SFM, sunflower meal; GH, grass hay; TTH, *Themeda triandra* hay; FM, fish meal.

**Table 2:** Ingredient and chemical composition of sunflower meal and fish meal concentrates used in Exp 5a and 5b

Items	Fish meal				Sunflower meal			
	
	16%	20%	24%	28%	16%	20%	24%	28%
Ingredient composition								
Maize (g/kg)	848	784	714	649	747	615	484	353
FM or SFM (g/kg)	103.9	170.3	238.2	303.1	205.5	336.9	468.2	599.5
Vit and minerals (g/kg)	2.53	2.53	2.53	2.53	2.53	2.53	2.53	2.53
Limestone (g/kg)	20.2	20.2	20.2	20.2	20.2	20.2	20.2	20.2
MCP (g/kg)	20.2	20.2	20.2	20.2	20.2	20.2	20.2	20.2
NaCl (g/kg)	5	5	5	5	5	5	5	5
Chemical composition								
Dry matter (g/kg)	880	882	887	889	890	895	895	904
Organic matter (g/kg)	926	911	903	896	939	925	914	912
Crude protein (g/kg)	149.5	189.3	239.7	277.4	151.1	191.4	231.3	271.1
NDF (g/kg)	81.8	80.5	111.1	111.7	145.2	182	225.7	269.3
ADF (g/kg)	81.1	80.4	97.3	97.6	116.0	136.3	160.4	184.4
Crude fat (g/kg)	44.7	43.2	48.4	55	48.4	51.4	54.6	57.9

FM, fish meal; SFM, sunflower meal; Vit, vitamins; MCP, monocalcium phosphate; NaCl, sodium chloride; NDF, neutral detergent fibre; ADF, acid detergent fibre.

**Table 3:** Effect of improving veld hay quality on diurnal feeding behaviour in Merino sheep (Exp 1, 3, 5) and Nguni goats (Exp 2)

Items	Intake (kg/d)		Rumination time (/d)		DM intake rate		BMC
			
	DM	NDF	min/kg DMI	min/kg NDFI	g/min	g/bout	kg/d
Experiment 1							
IRQ	1.55^a^	1.16^a^	318^b^	426^b^	5.8^a^	148^a^	−0.02^a^
PRQ	1.10^b^	0.81^b^	597^a^	813^a^	6.1^a^	119^a^	−0.16^b^
SEM	0.052	0.039	24.38	33.06	0.641	11.97	0.026
Significance	**	**	***	***	NS	NS	*
Experiment 2							
IRQ	0.92	0.83	421	466	3.2	65	−0.012^a^
SIRQ	0.89	0.81	390	424	3.1	72	−0.032^a^
PRQ	0.63	0.58	513	556	2.6	55	−0.071^b^
SEM	0.111	0.101	59.85	65.07	0.4185	9.271	0.0094
Significance	NS	NS	NS	NS	NS	NS	***
Experiment 3							
100% PRQ	1.09	0.94	546	636	2.97	56.2	ND
75% PRQ+25% LH	1.25	1.04	492	592	3.90	72.7	ND
50% PRQ+50% LH	1.41	1.13	442	550	4.59	73.6	ND
25% PRQ+75% LH	1.37	1.06	502	648	5.42	76.3	ND
100% LH	1.59	1.19	370	496	6.20	90.6	ND
SEM	0.229	0.180	70.62	88.91	0.8707	13.76	ND
Significance	NS	NS	NS	NS	NS	NS	ND
Experiment 5a							
TTH+16% FM	0.91	0.37	546	1,339	4.24	50.0	0.174
TTH+20% FM	0.92	0.37	619	1,520	4.01	50.8	0.199
TTH+24% FM	0.92	0.39	527	1,248	4.13	50.6	0.180
TTH+28% FM	0.89	0.37	624	1,477	3.74	49.7	0.188
SEM	0.020	0.008	29.91	71.52	0.3104	1.908	0.021
Significance	NS	NS	NS	NS	NS	NS	NS
Experiment 5b							
TTH+16% SFM	0.90	0.39^b^	550	1,253	4.12	49.7	0.163^a^
TTH+20% SFM	0.92	0.42^a^	522	1,141	4.18	51.6	0.138^a^
TTH+24% SFM	0.94	0.44^a^	489	1,043	4.65	52.1	0.145^a^
TTH+28% SFM	0.90	0.45^a^	531	1,060	4.20	49.6	0.096^b^
SEM	0.019	0.009	29.98	65.65	0.4476	1.790	0.015
Significance	NS	***	NS	NS	NS	NS	*

DM, dry matter; BMC, body mass change; NDF, neutral detergent fibre; DMI, dry matter intake; NDFI, neutral detergent fibre intake; IRQ, improved roughage quality; PRQ, poor roughage quality; SEM, standard error of the mean; NS, not significant; SIRQ, semi-improved roughage quality; ND, not determined; LH, lucerne hay; TTH, *Themeda triandra* hay; FM, fish meal; SFM, sunflower meal.

a,b Means in a column with different superscripts are significantly different (p<0.05).

* p<0.05;

** p<0.01;

*** p<0.001.

**Table 4:** Effect of improving veld hay quality on duration of day-time and night-time feeding behaviour patterns in Merino sheep (Exp 1)

Behaviour	Feeds						SEM	Significance of influence		

	IRQ			PRQ						
		
	Day	Night	24 h period	Day	Night	24 h period		Feed	Period	Feed×period
Time spent (min)										
Eating	222	52	274^a^	140	47	187^b^	12.56	*	***	*
Ruminating	188	305	493^b^	230	424	654^a^	5.131	***	***	***
Chewing	410	357	767^b^	370	471	841^a^	11.60	*	NS	***
Idling – standing1)	112	87	199	169	93	262	14.27	NS	**	NS
Idling – lying1)	198	277	475^a^	185	152	338^b^	24.32	*	NS	*
Duration of bouts (min)										
Eating	28	20	26	20	19	21	2.886	NS	NS	NS
Ruminating	20	23	22^b^	25	37	32^a^	1.763	***	***	*
Number of bouts										
Eating	8	3	11	7	2	9	0.695	NS	***	NS
Ruminating	10	13	23	10	12	22	1.016	NS	*	NS

IRQ, improved roughage quality; PRQ, poor roughage quality; SEM, standard error of the mean; NS, not significant.

1) Idling; any period of time when the animal is not ruminating or eating, and includes behaviours such as licking, fighting, drinking water, scratching and sleeping

a,b Means in a row with different superscripts are significantly different (p<0.05).

* p<0.05;

** p<0.01;

*** p<0.001.

**Table 5:** Effect of improving veld hay quality on duration of day-time and night-time feeding behaviour patterns in Nguni goats (Exp 2)

Behaviour	Feeds									SEM	Significance of influence		

	IRQ			SIRQ			PRQ						
			
	Day	Night	24 h	Day	Night	24 h	Day	Night	24 h		Feed	Period	F×P
Time spent (min)													
Eating	216	76	292	237	58	295	193	56	249	16.29	NS	***	NS
Ruminating	112	291	403	89	269	358	70	227	297	26.95	NS	***	NS
Chewing	328	366	694	326	327	653	263	283	546	29.07	NS	NS	NS
Idling – standing1)	158	54	212	166	46	212	208	53	261	15.84	NS	***	NS
Idling – lying1)	231	300	531	225	347	572	246	384	630	32.21	NS	**	NS
Duration of bouts (min)													
Eating	22	18	21	26	18	25	22	19	21	1.306	NS	***	NS
Ruminating	24	29	27	23	27	26	16	25	23	2.00	*	**	NS
Number of bouts													
Eating	10	4	14	9	3	12	9	3	12	0.612	NS	***	NS
Ruminating	5	10	15	4	10	14	4	9	13	0.857	NS	***	NS

IRQ, improved roughage quality; SIRQ, semi-improved roughage quality; PRQ, poor roughage quality; F×P, feed×period interactions; SEM, standard error of the mean; NS, not significant.

1) Idling: any period of time when the animal is not ruminating or eating, and includes behaviours such as licking, fighting, drinking water, scratching and sleeping

* p<0.05;

** p<0.01;

*** p<0.001.

**Table 6:** Effect of varying veld hay to lucerne hay ratios on duration of day-time and night-time feeding behaviour patterns in Merino sheep (Exp 3)

Behaviour	Diets														SEM	Significance of influence		

	100% PRQ			75% PRQ + 25% LH			50% PRQ + 50% LH			25% PRQ + 75% LH			100% LH					
					
	Day	Night	24 h	Day	Night	24 h	Day	Night	24 h	Day	Night	24 h	Day	Night		Diet	Period	D×P
TSE (min)	225	142	367	193	135	328	180	122	302	148	108	256	158	110	12.34	***	***	NS
TSR (min)	282	276	558	314	285	599	312	237	549	314	265	579	263	269	13.86	NS	**	*
TSC (min)	508	418	926	507	419	926	491	359	850	462	373	835	421	379	14.38	***	***	NS
TSIS (min)	82	54	136	73	55	128	98	68	166	120	83	203	143	94	12.70	***	***	NS
TSIL (min)	130	248	378	139	246	385	131	293	424	138	264	402	157	248	18.96	NS	***	NS
DEB (min)	22	15	19	23	16	19	19	13	16	15	13	13	15	14	1.431	***	***	NS
DRB (min)	26	23	24	29	24	27	26	20	23	27	23	24	22	23	1.3	*	***	*
NEB	10	9	19	9	8	17	10	9	19	10	9	19	10	8	0.67	NS	*	NS
NRB	11	12	23	11	12	22	12	12	24	12	12	24	12	12	0.6	NS	*	*

PRQ, poor roughage quality; LH, lucerne hay; SEM, standard error of the mean; D×P, diet×period interactions; TSE, time spent eating; NS, not significant; TSR, time spent ruminating; TSC, time spent chewing; TSIS, time spent idling whilst standing; TSIL, time spent idling whilst lying; DEB, duration of eating bouts; DRB, duration of ruminating bouts; NEB, number of eating bouts; NRB, number of ruminating bouts.

* p<0.05;

** p<0.01;

*** p<0.001.

**Table 7:** Effect of varying levels of protein supplementation using lespedeza and sunflower meal on duration of 10 h day-time feeding behaviour patterns in Damara sheep (Exp 4)

Behaviour	Diets				SEM	Significance
	
	60% MSM+40% SFM	60% MSM+ 40% LSP	40% MSD+60% SFM	60% GH+40% SFM		p value
Time spent (min)						
Eating	219^ab^	290^a^	156^b^	189^ab^	25.58	*
Ruminating	131	163	115	140	14.49	NS
Chewing	350^b^	454^a^	271^c^	330^b^	15.24	***
Idling – standing1)	77	43	139	78	20.21	*
Idling – lying1)	174^a^	104^b^	190^a^	193^a^	16.34	*
Duration of bouts (min)						
Eating	11^b^	15^a^	9^b^	11^b^	0.75	**
Ruminating	6^b^	9^a^	6^b^	7^ab^	0.64	*
Number of bouts						
Eating	6	7	6	6	0.40	NS
Ruminating	6	6	6	7	0.58	NS

MSM, maize stover at milk stage; SFM, sunflower meal; LSP, lespedeza; MSD, maize stover at dry stage; GH, grass hay; SEM, standard error of the mean; NS, not significant.

1) Idling: any period of time when the animal is not ruminating or eating, and includes behaviours such as licking, fighting, drinking water, scratching and sleeping.

a,b Means in a row with different superscripts are significantly different (p<0.05).

* p<0.05;

** p<0.01;

*** p<0.001.

**Table 8:** Effect of different inclusion levels of fish meal and sunflower meal on duration of day-time and night-time feeding behaviour patterns in Merino sheep (Exp 5)

Diets	Period	Behaviour (min)							

		TSE	TSR	TSC	TSI	DEB	DRB	NEB	NRB
Experiment 5a									
TTH+16% FM	Day	154	219	373	346	19	15	9	16
	Night	66	276	342	376	8	19	9	9
	24 h	220	495	715	722	12	19	18	25
TTH+20% FM	Day	180	233	414	305	23	16	9	15
	Night	57	333	390	329	6	23	10	10
	24 h	237	566	804	634	12	23	19	25
TTH+24% FM	Day	164	198	362	357	20	14	9	15
	Night	66	286	352	367	8	19	9	9
	24 h	230	484	714	724	13	20	18	24
TTH+28% FM	Day	186	215	400	318	23	15	9	15
	Night	62	333	395	323	8	23	9	9
	24 h	248	548	795	641	14	23	18	24
Significance	SEM	9.65	14.35	18.31	18.28	2.58	1.70	0.99	0.99
	Diet	NS	*	*	*	NS	NS	NS	NS
	Period	***	***	NS	NS	***	***	NS	***
	Diet×period	NS	NS	NS	NS	NS	NS	NS	NS
Experiment 5b									
TTH+16% SFM	Day	156	198	354	365	19	14	9	15
	Night	65	293	358	361	8	19	9	9
	24 h	221	491	712	726	12	35	18	14
TTH+20% SFM	Day	168	187	355	364	21	12	9	16
	Night	57	289	347	372	7	20	9	9
	24 h	225	476	702	736	13	19	18	25
TTH+24% SFM	Day	168	184	352	367	20	13	9	15
	Night	60	276	336	383	8	18	9	9
	24 h	228	460	688	750	13	19	18	24
TTH+28% SFM	Day	168	183	352	367	20	13	10	14
	Night	63	293	356	362	8	18	9	9
	24 h	231	476	708	729	12	21	19	23
Significance	SEM	11.91	14.81	18.95	18.92	2.26	1.27	1.025	0.99
	Diet	NS	NS	NS	NS	NS	NS	NS	NS
	Period	***	***	NS	NS	***	***	NS	***
	Diet×period	NS	NS	NS	NS	NS	NS	NS	NS

TSE, time spent eating; TSR, time spent ruminating; TSC, time spent chewing; TSI, time spent idling; DEB, duration of eating bouts; DRB, duration of ruminating bouts; NEB, number of eating bouts; NRB, number of ruminating bouts; TTH, *Themeda triandra* hay; FM, fish meal; SEM, standard error of the mean; NS, not significant; SFM sunflower meal.

* p<0.20;

** p<0.15;

*** p<0.05.

**Table 9:** Effects of ruminant species, period of the day and their interactions on feeding behaviour (LSM±SEM) of sheep and goats fed varying roughage qualities in 6 different studies

Items	Feeding behaviour (min)						Significance				
	
	Goats			Sheep			Random effects		Fixed effects		
			
	D	N	Average	D	N	Average	ST×RQ	S	P	S×P	CP
TSE	206.4±24.9	54.4±24.9	130.4±22.0	179.2±13.0	82.6±13.2	130.9±12.6	*	NS	***	***	**
TSR	68.8±63.0	240.8±63.0	154.7±48.0	209.7±16.3	311.9±17.2	260.8±15.2	*	***	***	NS	**
TSC	269.7±77.1	289.3±77.1	279.5±56.2	408.6±17.5	309.3±19.4	359.0±13.5	NS	*	NS	NS	***
DEB	20.9±2.9	15.9±2.9	18.4±2.8	19.2±1.9	12.0±1.9	15.6±1.8	*	NS	***	NS	***
DRB	20.0±5.2	26.0±5.2	23.0±4.9	18.8±3.0	21.0±3.1	19.9±3.0	**	NS	***	NS	***
NEB	9.3±1.5	3.3±1.5	6.3±1.5	7.5±0.9	6.1±1.0	6.8±0.9	**	NS	***	***	NS
NRB	4.7±2.1	10.2±2.1	7.4±1.9	10.4±1.2	10.3±1.2	10.4±1.2	**	*	***	***	NS

LSM±SEM, least square means±standard error of the mean; D, day; N, night; ST×RQ, study by roughage quality interactions; S, species; P, period of day; S×P, species by period of day interactions; CP, crude protein; TSE, time spent eating; NS, not significant; TSR, time spent ruminating; DEB, duration of eating bouts; DRB, duration of ruminating bouts; NEB, number of eating bouts; NRB, number of ruminating bouts.

* p<0.20;

** p<0.15;

*** p<0.05.

**Table 10:** Effects of ruminant species on feeding behaviour (LSM±SEM) of sheep and goats fed varying diets and roughage qualities in 6 different studies

Items	Feeding behaviour		Significance		
	
	Ruminant species		Random effects	Fixed effects	
		
	Goats	Sheep	ST×RQ	S	CP
DMI (kg/d)	0.8±0.2	1.2±0.1	**	*	NS
NDFI (kg/d)	0.8±0.3	0.8±0.2	**	NS	NS
RTDMI (min/kg)	422.8±94.8	481.4±56.1	**	NS	NS
RTNDFI (min/kg)	441.1±234	879.5±174	*	*	NS
IR (g/min)	3.1±0.6	5.0±0.4	*	***	NS
IR (g/bout)	64.6±27.0	97.0±19.2	**	NS	NS

LSM±SEM, least square means±standard error of the mean; ST×RQ, study by roughage quality interactions; S, species; CP, crude protein; DMI, dry matter intake; NS, not significant; NDFI, neutral detergent fibre intake; RTDMI, rumination time per unit dry matter intake; RTNDFI, rumination time per unit neutral detergent fibre intake; IR, intake rate.

* p<0.20;

** p<0.15;

*** p<0.05.

**Table 11:** Pearson correlation of feed attributes and feeding behaviour parameters for all experimental data1)

Items	TSR	TSE	TSC	IRgmin	IRgbout	DM	CP	NDF	ADF	HEM
DMI	0.47***	0.21*	0.50***	0.70***	0.77***	−0.11^NS^	0.11^NS^	0.33***	0.28**	−0.03^NS^
TSR	-	0.10^NS^	0.89***	0.32***	0.29**	−0.24**	0.25**	−0.07^NS^	0.15^NS^	0.25392**
TSE	-	-	0.54***	−0.50***	0.09^NS^	0.09^NS^	−0.28**	0.48***	0.48***	−0.22*
TSC	-	-	-	0.05^NS^	0.29**	−0.16^NS^	0.08^NS^	0.16^NS^	0.09^NS^	0.11^NS^
IRgmin	-	-	-	-	0.59***	−0.11^NS^	0.27**	−0.01^NS^	−0.08^NS^	0.16^NS^
IRgbout	-	-	-	-	-	0.30**	−0.21*	0.50***	0.38***	0.06^NS^
DM	-	-	-	-	-	-	−0.56***	0.47***	0.25**	0.38***
CP	-	-	-	-	-	-	-	−0.64***	−0.61***	0.23*
NDF	-	-	-	-	-	-	-	-	0.94***	−0.34***
ADF	-	-	-	-	-	-	-	-	-	−0.62***
HEM	-	-	-	-	-	-	-	-	-	-

TSR, time spent ruminating; TSE, time spent eating; TSC, time spent chewing; IRgmin, intake rate g/min; IRgbout, intake rate g/bout; DM, dry matter; CP, crude protein; NDF, neutral detergent fibre; ADF, acid detergent fibre; HEM, hemicellulose; NS, not significant.

1) n = 114 and 22 diets.

* p<0.05;

** p<0.01;

*** p<0.001.
